# Authorship in studies conducted in low-and-middle income countries and published by *Reproductive Health*: advancing equitable global health research collaborations

**DOI:** 10.1186/s12978-020-0858-7

**Published:** 2020-01-30

**Authors:** Verónica Pingray, Vanesa Ortega, Sanni Yaya, José M. Belizán

**Affiliations:** 1 Department of Mother and Child Health Research, Institute for Clinical Effectiveness and Health Policy (IECS-CONICET), Buenos Aires, Argentina; 2 The George Institute for Global Health, The University of Oxford, Oxford, UK; 3 School of International Development and Global Studies, Faculty of Social Sciences, University of Ottawa, Ottawa, Canada

*Reproductive Health* has an interest in reproductive health status globally, but it has particular interest in phenomena affecting disadvantaged populations. This is the reason why this journal encourages submissions from researchers conducting studies in low- and middle-income countries (LMICs).

Authorship usually reflects research leadership [1]. At the same time, authorship implies responsibility and accountability for published work [2]. In addition, authorship has important academic, social, and financial implications, particularly for the first, second and last authors. In the context of global health research, authorship might also indicate the level of balance within collaborative research and the success of capacity-building [1].

Researchers who have made substantive contributions to a study or a paper should receive credit as authors. The International Committee of Medical Journal Editors (ICMJE) recommends that authorship is based on the following criteria: a) substantial contributions to the conception, design, acquisition, analysis, or interpretation of data; b) drafting the work or revising it critically for important intellectual content; c) provide the final approval of the version to be published; and d) agree to be accountable for all aspects of the work [2]. These authorship criteria are intended to reserve the status of authorship for those who deserve credit and can take responsibility for the work. ICMJE recommends sharing co-authorship with colleagues in the locations where the research is conducted. However, the ICMJE does not establish criteria to determine the order in which authors are listed. Such ordering involves differential credits, and it should be decided on collectively by the research team [2].

We performed a descriptive analysis of all research articles published during 2018 in *Reproductive Health* in order to: a) describe the location where studies were conducted; b) determine the location of authors’ affiliation in studies conducted in LMICs; and c) explore the type of credit that researchers from LMICs received in collaborative research.

During 2018, 219 articles were published in *Reproductive Health*. After excluding study protocols, reviews, commentaries, editorials and corrections, 157 research articles were included (Additional file 1: Figure S1). Among research studies, 123 (81%) were conducted in at least one LMIC. (Table 1) The region with the largest number of published studies was Sub-Saharan Africa (*n* = 81, 53%) (Fig. 1). All the other regions of the world published at least 5 times fewer studies. Overall, 133 studies were conducted in 47 single countries, and 19 studies were conducted in two or more countries.

**Table 1:** Countries where published studies in Reproductive Health during 2018 were conducted

Study location	Number of studies (n) *N* = 152	Percentage (%)
*Income group by The World Bank*		
Low-income countries	55	36,2
Middle-income countries	60	39,5
High income-countries	29	19,1
More than one income group	8	5,3
*Country*		
More than one	19	12,5
Ethiopia	16	10,5
India	12	7,9
China	9	5,9
Tanzania	9	5,9
Kenya	7	4,6
Ghana	6	3,9
South Africa	6	3,9
Uganda	6	3,9
Australia	4	2,6
Malawi	4	2,6
USA	4	2,6
Brazil	3	2,0
Nigeria	3	2,0
Sierra Leone	3	2,0
Sudan	3	2,0
Burkina Faso	2	1,3
Congo	2	1,3
Mexico	2	1,3
Mozambique	2	1,3
Nepal	2	1,3
Zambia	2	1,3
Bangladesh	1	0,7
Cyprus	1	0,7
Czech Republic	1	0,7
Denmark	1	0,7
Egypt	1	0,7
Estonia	1	0,7
France	1	0,7
Gambia	1	0,7
Germany	1	0,7
Guatemala	1	0,7
Guinea	1	0,7
Iran	1	0,7
Iraq	1	0,7
Jordan	1	0,7
Kenya	1	0,7
Lebanon	1	0,7
Netherlands	1	0,7
Palestina	1	0,7
Philippines	1	0,7
Spain	1	0,7
Sri Lanka	1	0,7
Suiza	1	0,7
Tajikistan	1	0,7
Thailand	1	0,7
Turkey	1	0,7
Uruguay	1	0,7

**Fig. 1:**
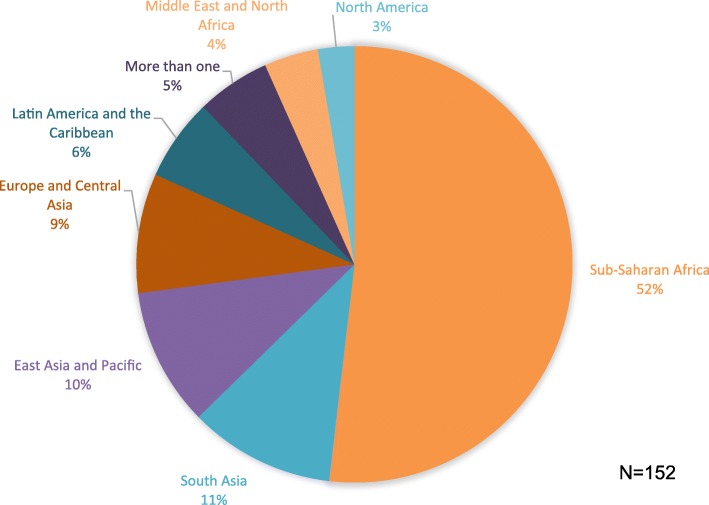
World regions where published studies in *Reproductive Health* in 2018 were conducted

Among the studies conducted in LMICs (*n* = 123), 86 (70%) were collaborations between authors affiliated to organizations from more than one income group (Table 2). Sixteen (13%) studies were conducted exclusively by local researchers from LICs, and 21 (17%) by researchers from MICs. Although collaborative studies were the most frequent type (70%), only 45 (40%) of the publications combined first, second and last authors (from different country income groups).

Analyzing only collaborative studies (*n* = 86), it was observed that 49 (57%) publications combine the origin of the first, second and last author (from different country income groups). In contrast, 24 (28%) of collaborative studies assigned first, second and last authorship to researchers from high-income countries, while 13 (15%) assigned these three positions to authors from low or middle-income countries.

**Table 2:** Authors´ affiliations of studies conducted in LMICs published in 2018 in *Reproductive Health* during 2018

	Authors´ affiliations n (%) *N* = 123	First, second and last author n (%) *N* = 123
All from Low-income countries	16 (13)	20 (16)
All from Middle-income countries	21 (17)	29 (24)
All from High-income countries	0	25 (20)
Combined/Mixed	86 (70)	49 (40)

Finally, we analyzed the distribution of first, second and last authors according to the income group of their affiliated country, in studies conducted in LMICs (*n* = 123) (Fig. 2). We observed that 51 (42%) of first authors were from HICs and 59 (48%) of last authors were from HICs, while second author position was more equally distributed among low-income, middle- income and high-income countries (29%, 38% and 34% respectively).

**Fig. 2:**
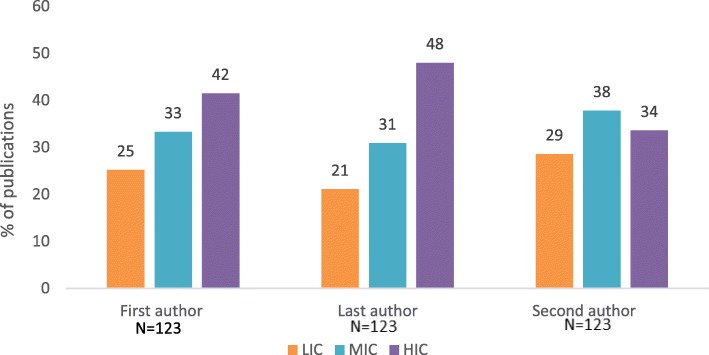
Authorship order and country of affiliation income group in studies conducted in LMICs and published by *Reproductive Health* in 2018

This analysis shows that most research studies published by *Reproductive Health* during 2018 were conducted in LMICs. At the same time, studies conducted in LMICs seemed to be more frequently conceived and driven by high-income-country authors. Although authorship tends to demonstrate some balance in collaborative research conducted in LMICs, first and last authorship are more frequently assigned to HIC researchers. About one third of collaborative research conducted in LMICs still has first, second and last authorship assigned to HIC researchers.

Multiple barriers might be influencing the observed results. First, LMICs usually present a disadvantaged and unequal position in terms of language fluency and writing skills that could affect publication success and their contribution during the development of manuscripts [3, 4]. Though *Reproductive Health* accepts abstracts and even full texts in languages other than English, the original manuscript--the one subjected to editor and reviewer evaluations--must be written in English. Despite the disadvantaged situation of researchers from LMICs, *Reproductive Health*, like most journals, has no capacity to offer free copyediting services or support for writing manuscripts. Second, there is low awareness of ICMJE guidelines among LMICs authors and low used of a structured application of the recommended authorship criteria [5–7].

Researchers affiliated to organizations in HICs have advantages in terms of obtaining funding, partially due to their affiliations’ credibility and publication background. Somehow, this could create a virtuous cycle for researchers affiliated to organizations in HICs, which might contribute to them receiving the required support for their research questions and plans.

There is a global responsibility when conducting collaborative research to include LMICs researchers in terms of recognizing the efforts and contributions made to complete work; as well as build capacity, in order to increase the possibilities of LMIC researchers being included in the virtuous cycle of receiving support to conduct studies locally.

Considering the unbalanced training, access to funding and publication, a local research capability plan should be part of collaborative work. Ideally, funding agencies committed to increasing research equality, should be encouraged to develop research capabilities plans. In such way, there is also a need for the development and maintenance of research centres in LMICs that encourage and strengthen multidisciplinary teams of researchers to conduct independent research [8]. This would be an investment to balance the funding of future research questions generated in both HICs and LMICs.

In addition, active dissemination of fee waivers should be promoted within open access journals for LMICs and collaborative academic programs that provide support to LMICs researchers.

Further research should be conducted to better explain trends and factors influencing authorship in studies conducted in LMICs, while, at the same time, the research community reflects on our active commitment to generating equitable collaborations in global research.

## Supplementary information

**Additional file 1: Figure S1.** Total number of publications in *Reproductive Health* during 2018 and number of included studies in this analysis.
