# Supplementary material for: Authorship in studies conducted in low-and-middle income countries and published by Reproductive Health: advancing equitable global health research collaborations
Source: Reprod Health. 2020 Jan 30;17:18. doi: 10.1186/s12978-020-0858-7 (PMC6993386; doi:10.1186/s12978-020-0858-7)
Supplement: Supplementary file 1 — Additional file 1: Figure S1. Total number of publications in Reproductive Health during 2018 and number of included studies in this analysis. [file 12978_2020_858_MOESM1_ESM.docx]

Supplementary figure 1: total number of publications in *Reproductive Health* during 2018 and number of included studies in this analysis
